# Exploring predictors of Treatment Attendance in Patients with PTSD and Comorbid Personality Disorders: Secondary Analysis of a Randomized Controlled Trial

**DOI:** 10.1192/j.eurpsy.2024.143

**Published:** 2024-08-27

**Authors:** A. van den End, A. Snoek, I. Aarts, N. Lommerse, J. Dekker, A. T. F. Beekman, K. Thomaes

**Affiliations:** ^1^Sinai Centre, Amstelveen; ^2^Psychiatry, Vrije Universiteit Medical Centre, Amsterdam; ^3^Psychiatry, Vrije Universiteit Medical Centre, Amstelveen; ^4^Anatomy and Neurosciences, Vrije Universiteit Medical Centre; ^5^Arkin; ^6^Clinical Psychology, Vrije Universiteit, Amsterdam; ^7^Arkin, Amstelveen, Netherlands

## Abstract

**Introduction:**

Posttraumatic stress disorder (PTSD) and personality disorders (PD) often co-occur and treatment dropout remains a challenging problem for both disorders. The literature on predictors of treatment dropout is highly mixed and few reliable predictors have been identified for both PTSD and PD treatments separately, let alone for concurrent PTSD and PD treatment.

**Objectives:**

The aim of the present study was to identify predictors of treatment attendance among a wide range of variables in patients with PTSD and comorbid PD who received trauma-focused treatment with and without concurrent PD treatment.

**Methods:**

Data were used from the prediction and outcome study in comorbid PTSD and personality disorders (PROSPER), a study consisting of two randomized clinical trials (RCT) testing the effectiveness of trauma-focused treatment (eye movement desensitization and reprocessing or imagery rescripting) with versus without concurrent PD treatment (dialectical behavior therapy or group schema therapy). 256 patients with PTSD and comorbid personality disorder participated in the study. The potential predictors included demographic (e.g. work status), patient severity (e.g. PTSD severity), patient-therapist (e.g. working alliance) and therapist (e.g. therapist experience) variables. The ordinal outcome variable was treatment attendance (0, 1-7, 8-11, 12+ trauma-focused treatment sessions). Relevant predictors were identified by a series of ordinal regression analyses (threshold for inclusion p < .10). Relevant predictors were then entered together in a final ordinal regression model. Multiple imputation was used to handle missing data.

**Results:**

The final model included ten predictor variables and provided a good fit for the data (pooled R^2^_Nagelkerke_ = .29). Higher education level (OR = 1.22, p = .009), self-rated PTSD severity (OR = 1.04, p = .036) and working alliance (OR = 1.72, p = .047) were associated with a larger number of attended sessions. Higher levels of inadequate social support from a friend (OR = 0.90, p = .042) and being randomized in the concurrent treatment condition (OR = 0.52, p = .022) were associated with a smaller number of attended sessions.

**Conclusions:**

In terms of treatment attendance rates, the results suggest that trauma-focused treatment is preferred over concurrent trauma-focused and personality disorder treatment for patients presenting with PTSD and PD. Clinicians should further be aware of the risk of lower treatment attendance for patients with a lower educational background and those reporting inadequate social support. Enhancing working alliance may protect against early treatment termination. Finally, patients with higher levels of PTSD severity at baseline may need a larger number of treatment sessions.

**Disclosure of Interest:**

None Declared

